# Clinical Practice: Giant Cell Tumour of the Jaw Mimicking Bone Malignancy on Three-Dimensional Computed Tomography (3D CT) Reconstruction

**DOI:** 10.2174/1874210600802010073

**Published:** 2008-06-03

**Authors:** Alessandro Lanza, Luigi Laino, Luigi Rossiello, Letizia Perillo, Antonio Dell Ermo, Nicola Cirillo

**Affiliations:** 1 Regional Center on Craniofacial Malformations-MRI, Section of Genetic Oral Diseases; 2 Department of Odontostomatology, and , 1st School of Medicine and Surgery, II University of Naples, 80138 Naples, Italy; 3 Department of Dermatology, 1st School of Medicine and Surgery, II University of Naples, 80138 Naples, Italy; 4 Department of Experimental Medicine, 1st School of Medicine and Surgery, II University of Naples, 80138 Naples, Italy

## Abstract

A wide range of diseases may present with radiographic features of osteolysis. Periapical inflammation, cysts and benign tumours, bone malignancies, all of these conditions may show bone resorption on radiograph. Features of the surrounding bone, margins of the lesion, and biological behaviour including tendency to infiltration and root resorption, may represent important criteria for distinguishing benign tumours from their malign counterpart, although the radiographic aspect of the lesion is not always predictive. Therefore a critical differential diagnosis has to be reached to choose the best management. Here, we report a case of giant cell tumour (GCT) whose radiological features by computed tomography (CT) suggested the presence of bone malignancy, whereas the evaluation of a routine OPT scan comforted us about the benign nature of the lesion. A brief review of the literature on such a benign but locally aggressive neoplasm is also provided.

## INTRODUCTION

Giant cell tumors (GCTs) chiefly occur at the ends of long bones. When they occur in unusual sites, such as the head and neck region, it may be difficult to make a correct preoperative diagnosis. Nevertheless, it is important to clear the biological nature of the lesion before surgery.

## CASE PRESENTATION

**Fig. (1):**
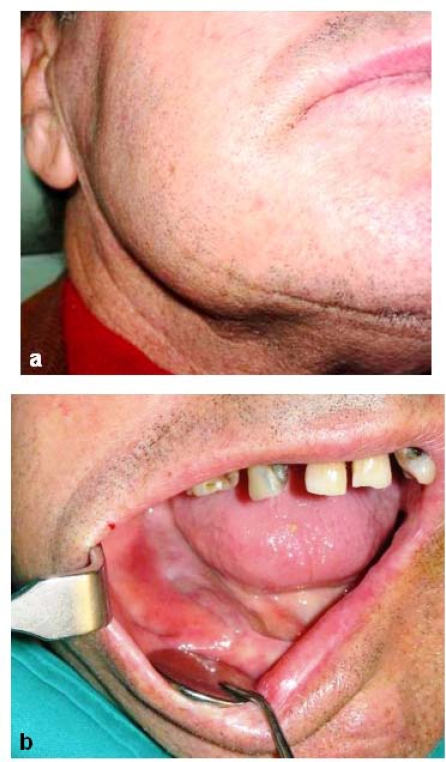
**(a) **Extraoral examination shows swelling of the jaw causing facial asymmetry.** (b) **Red-purple prominent lesion involving alveolar crest that appears swollen, edematous and eroded, causing deformity of the jaw.

**Fig. (2):**
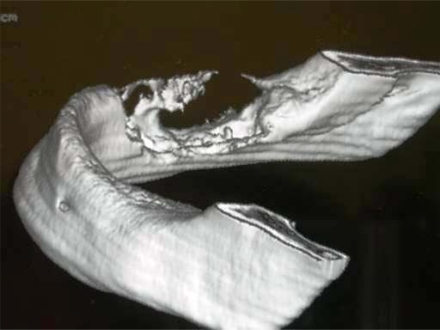
3D CT reconstruction of the internal (lingual) aspect of the jaw demonstrates massive destruction of the lingual cortical bone; vestibular cortical bone appears as an interrupted thin line. The three-dimensional picture points out loosening of the trabecular features of the jaw bone.

**Fig. (3):**
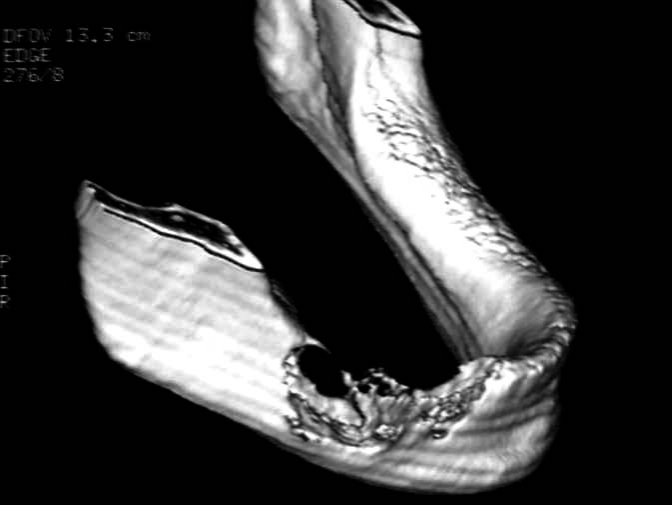
3D CT of the external aspect of the jaw shows an apparently infiltrative nature of the lesion with invasive margins involving contiguous healthy bone.

A 77-year-old male was referred by his dentist to the Odontostomatology service at the Second University of Naples. The patient complained of a painful swelling at the right inferior jaw that caused facial asymmetry (Fig. **1a**). The swelling had increased in size in the last two months. However, the patient did not refer any motor or sensory deficit. Intraorally, the red-purple prominent mass causing swelling involved the alveolar crest that appeared edematous and eroded (Fig. **1b**). Neck lymphonodes were palpable and painful. The results of the blood chemistry and routine laboratory tests were normal. Anamnesis revealed that antibiotic therapy had been prescribed by his dentist, after which the patient referred a regression of pain not accompanied by reduction of swelling. Therefore, an orthopantomography (OPT) and computed tomography (CT) scans had been taken. Unfortunately, the patient had lost his OPT. The three-dimensional (3D) reconstruction of tomography showed a wide osteolytic area with massive bone destruction and partial perforation of the cortical bone (Fig. **2** and Fig. **3**). Margins were irregular and the lesion seemed to infiltrate the surrounding tissue.

## DIFFERENTIAL DIAGNOSIS

*Dr. Alessandro Lanza.* The clinical and radiographic features are poor and do not allow to draw strong diagnostic conclusions. Differential diagnosis should be established among brown tumours and fibroosseous lesions, keratocystic odontogenic tumor, and malignant neoplasm of the jawbone such as sarcomas and Langerhans cell histiocytosis. Such irregular margins of osteolysis revealed by CT would suggest malignancy, although locally invasive neoplasm such as giant cell tumour and keratocystic odontogenic tumor can not be excluded. Fine needle aspiration may be useful for ascertaining cystic nature (fluid content)****[1]. I suggest explorative surgery and biopsy to clarify the nature of the lesion and manage it accordingly [2].

*Dr. Nicola Cirillo.* Thinning or perforation of the cortex associated with a soft tissue mass, rapid invasion of mucosal tissues causing swelling along with jaw osteolysis with pretty irregular margins would be suggestive of aggressive lesions such as sarcomas or Langerhans cell histiocytosis [3]. On the other hand, localized swelling is an important clinical feature of Giant cell granuloma that is usually painless and remains undetected until facial asymmetry. Although sensory deficits typical of osteosarcoma were not present in our patient, giant cell tumour and keratocystic odontogenic tumor could mimic malignancies because of their locally invasive features [4]. Indeed, margins may be difficult to distinguish because of the high infiltrative nature of these tumours. In addition, 3D reconstruction imaging of CT may be confusing, whereas a gross radiographic examination such as OPT would be helpful. Patient should be screened for both hypercalcemia and hyperparathyroidism to differentiate the lesion from brown tumors. I recommend bone biopsy.

## MANAGEMENT 

**Fig. (4):**
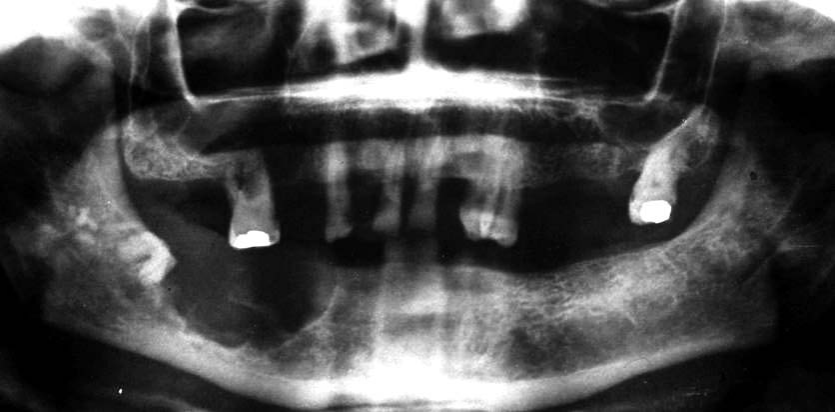
The OPT suggests a locally aggressive lesion with defined margins and it excludes the possibility of a true malignancy of the bone.

**Fig. (5):**
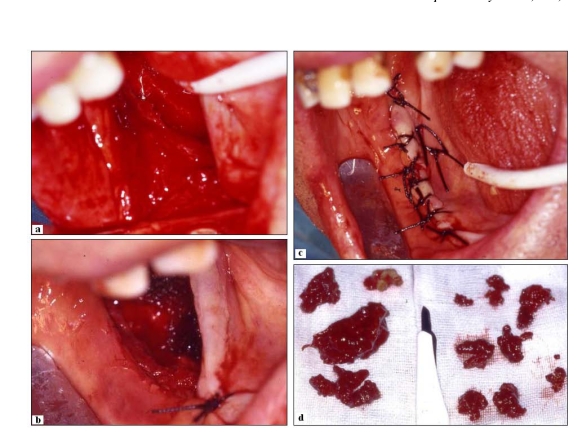
**(a-b)  **Intraoperative phases.  ** (c)** Suture (interrupted type) of the surgical wound; ** (d)**  Macroscopical aspect of the lesion excised. It exhibits typical features of giant cell tumour, including brown appearance.

**Fig. (6):**
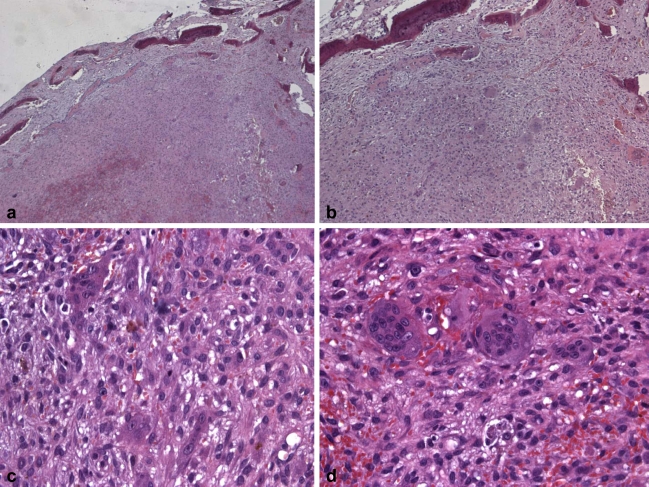
Histological features of the tissue stained with haematoxylin-eosin. ** (a) ** Low magnification (E-E, 25X) figure showing granulation tissue and bone islets. ** (a) ** Higher magnification (E-E, 50X) of the previous slide.  **(c) ** Tissue section illustrating the detailed histomorphology of the lesion (E-E, 200X).  **(d) ** High resolution image (E-E, 400X) of multinucleated giant cells.

Before surgery, an OPT was requested that suggested benign nature of the lesion (Fig. **4**). The patient underwent excision and curettage of the mass. The curetted reddish, brown material is shown in Fig. (**5**). The wound was closed with interrupted sutures (Fig. **5a**). Enough inferior and lingual cortex remained to provide adequate mandibular stability. Thus, no internal fixation or plating was needed. The histopathological examination revealed multinuclear giant cells scattered randomly throughout the cellular and fibrous vascular-rich tissue (Fig. **6**). New bone formation and granulation tissue rich in mononuclear inflammatory cells was revealed (Fig. **6a,6b**). The giant cells were multinucleated with bland-appearing nuclei, and the background stromal cells displayed mild to moderate atypia with no evidence of atypical mitoses (Fig. **6c,d**). A diagnosis of giant cell tumour was established.

## DISCUSSION

GCT is a benign but locally aggressive neoplasm and represents 4 to 9.5% of all primary osseous neoplasm [6]. It affects all races with a particular prevalence in the oriental populations. Patients most affected are comprised between 20 and 40 years old with a high prevalence for middle aged patients and older patients. GCT exhibits a slight predominance for the female sex [7]. It is characterized by infiltrative nature and unpredictable biological behavior with high tendency to recurrences. CGT with major aggressive behavior and high recurrences are those potentially causing metastasis that appear very rarely, approximately in 1-9% of CGT of bone and affecting lungs [8]. Malignant transformation of a previous benign CGT was described in literature after radiation therapy [9]. Surgery is the main treatment modality for giant cell tumors of bone and includes curettage alone, curettage combined with adjuvant therapy (cryosurgery and bone cement or bone graft), bone resection and amputation.

GCT shows many clinical and radiological features in common with other bony lesion of the jaw, including giant cell granuloma, aneurismal bone cyst, fibroosseous lesions, cherubism, odontogenic myxoma, vascular lesions of the bone, keratocystic odontogenic tumor, cystic ameloblastoma and malignant neoplasm of the jawbone such as sarcoma and Langerhans cell histiocytosis [10]. An early diagnosis is crucial to avoid serious complications. Therefore, it becomes essential that radiologists and clinicians do identify accurately both radiographic and clinical features, extension of the lesion and especially its biological nature.

Cherubism, CGT, Brown tumour of mandible and Central giant cell granuloma (CGCG) are different disease but with similar clinical features and/or similar radiographic aspects and all composed by multinucleated giant cells.

### Cherubism

Cherubism is a genetically determined non-neoplastic bone disease characterized by bilateral painless swelling of the jaw affecting children from 2 to 4 years and more rarely young adults. It has no predominance of gender [11]. The bilateral involvement of the mandible and the typical facies of cherubism patients, along with the presence of familiarity in association with the particular prevalence in the young population, are major clinical features essential for clinicians to distinguish between cherubism and CGT [12].

### Giant Cell Granuloma

Giant cell granuloma is a bony lesion more common in the jaw than in other bony sights of the body. It represents the 7% of all benign lesions of the jaw. Many pathologists consider CGCG as a reparative process following a trauma others as a true neoplasm. CGCG present clinical and radiographic features overlapping GCT. Some criteria useful to differentiate the two lesions may be: more infiltrative nature of the CGT, and different prevalence of the CGCG, which affects younger adults with age comprised between 3 and 17 (or, generally, patients less than 30 years-old). There are no true differences in radiographic and clinical aspects between the two lesions [13]. True CGT is composed by multinucleated cells with a larger number of nuclei centrally located and indistinguishable from nuclei of stromal cells. Therefore pathology is essential to distinguish the two lesions [14].

### Brown Tumours

Brown tumours of the mandible are focal lesions of the mandible radiographically and clinically undistinguishable from other giant cell lesions [15]. Brown tumors have traditionally been associated with hyperparathyroidism, which causes imbalance in osteoclastic-osteoblastic homeostasis. Thus, an important criterion essential to make a differential diagnosis is the research of hyperparathyroidism and hypercalcemia, alteration in calcium-phosphorous balance and increase of parathyroid hormone (PTH) in blood chemistry. None of these features was found in our patient [16].

CGT shares many features also with vascular lesions of the bone such as ***Aneurysmal bone cyst***. It is important for these cysts to be distinguished from CGT and other jawbone tumours. Unfortunately, typical clinical aspects as swelling and pain which usually follows an injury and radiographic characteristics showing an outlined, bulging, destructive and eccentric lesion of the bone are not sufficient to make a differential diagnosis [17]. However, aneurysmal bone cyst is typically composed of honeycomb blood-filled spaces with a lining of flat nonendothelial cells. Therefore, biopsy is usually required [18].

### Non-Ossifying Fibroma

Non-ossifying fibroma has similar clinical and radiographic aspects to the GCG. Differential diagnosis between the two lesions is based on biopsy [19]. Main histological features of CGT such as aggregate of giant cells and particular fibrous stroma are not typical aspects of non-ossifying fibroma [20].

### Osteosarcoma

Osteosarcoma is the most common primary sarcoma involving the jawbones. Pain and swelling are the most common symptoms. Radiographically advanced cases of osteosarcomas may demonstrate a poorly defined osteolytic, lesions, localized supereruption of a tooth, a “floating tooth” or a ‘‘hanging tooth’’ caused by resorption of bone around the root and bone formation above the alveolar crest [21]. CT typically reveals cortical destruction, periosteal reaction and soft tissue and intramedullary bone involvement. Peripheral margins are usually poorly defined. In the case reported here, all these features were detectable in our 3D reconstruction, along with rapid growth and lymphadenopathy. On the contrary, the OPT showed less infiltrative nature. In general, final diagnosis is based upon hematoxylin and eosin microscopy taken in an appropriate clinical context. Osteosarcomas require the finding of atypical cells in association with immature, haphazardly distributed osteoid and the absence of giant cell with large multiple nuclei inside of a true CGT [22].

Surgery is the traditional and most accepted form of treatment for GCTs that include simple excision and curettage or partial jaw resection “en bloc”. Combination with adjuvant treatment, such as the use of phenol and methylmethacrylate, reduces the incidence of recurrence to less than 10% [23]. The local recurrence has been shown to correlate with aggressiveness of the lesion. The incidence of recurrence after surgery is 4-20%, whereas locally aggressive giant cell lesions have a higher recurrence rate.

GCTs have also been treated by non-surgical methods which include principally radiotherapy. Some GCT may also be sterilized thermally, using laser or cryoprobe. Much controversy still exists, in the literature, concerning radiation treatment, which has been reported as a potential risk for malignant transformation [24].

In conclusion, here we have provided an exemplificative case of bone osteolysis of uncertain origin and reviewed the criteria to be adopted in making a correct diagnosis.
